# Epinephrine reversed high-concentration bupivacaine- induced inhibition of calcium channels and transient outward potassium current channels, but not on sodium channel in ventricular myocytes of rats

**DOI:** 10.1186/s12871-015-0049-1

**Published:** 2015-04-30

**Authors:** Fuli Liu, Bingjing Wu, Yongjun Du, Yiquan Wu, Hongfei Chen, Fangfang Xia, Zhousheng Jin, Xuzhong Xu

**Affiliations:** 1 Department of Anesthesiology, the First Affiliated Hospital of Wenzhou Medical University, 2 Fuxue Road, 325000 Zhejiang, China; 2 Environment and health institute, Wenzhou Medical University, Zhejiang, China

**Keywords:** Bupivacaine, Epinephrine, Cardiac toxicity

## Abstract

**Background:**

Epinephrine is a first-line drug for cardiopulmonary resuscitation, but its efficacy in the treatment of bupivacaine-induced cardiac toxicity is still in question. We hypothesized that epinephrine can reverse cardiac inhibition of bupivacaine by modulating ion flows through the ventricular myocyte membrane channels of rats. The aim of this study was to observe and report the effects of epinephrine on high-concentration bupivacaine-induced inhibition of sodium (I_Na_), L-type calcium (I_Ca-L_), and transient outward potassium (I_to_) currents in the ventricular myocytes of rats.

**Methods:**

The ventricular myocytes were isolated from Sprague-Dawley rats (250-300 g) by acute enzymatic dissociation. The whole-cell patch clamp technique was used to record the ion channel currents in single ventricular myocytes both before and after administration of medications.

**Result:**

Administration of bupivacaine 100 μmol/L significantly reduced I_Na_, (P < 0.05). However, administration of bupivacaine 100 μmol/L in conjunction with epinephrine 0.15 μg/ml had no effect in restoring I_Na_ to its previous state. Similarly, a sharp decline of I_Ca-L_ and I_to_ was observed after administration of bupivacaine 100 μmol/L (P < 0.05). In contrast to I_Na_, I_Ca-L_ and I_to_ were significantly improved after the administration of the aforementioned combination of bupivacaine and epinephrine (P < 0.05).

**Conclusion:**

Epinephrine can reverse high-concentration bupivacaine induced inhibition of I_Ca-L_ and I_to_, but not I_Na_. Thus, epinephrine’s effectiveness in reversal of bupivacaine-induced cardiac toxicity secondary to sodium channel inhibition may be limited.

## Background

Epinephrine is a first-line drug for cardiopulmonary resuscitation. However, the extent of its effectiveness in bupivacaine-induced cardiac toxicity has remained unresolved [1-4]. Weinberg et al. [1] and Hiler et al. [2] reported that using epinephrine for resuscitation may increase myocardial oxygen consumption in the bupivacaine intoxication model of rats and rabbits. Its use in this setting may result in refractory ventricular fibrillation, pulmonary edema, acidosis, hypoxemia and other complications. On the other hand, Harvey et al. [3] claimed that epinephrine was necessary for circulatory recovery in their rabbit model of bupivacaine toxicity. Their claim was supported by work that demonstrated that epinephrine can accelerate hemodynamic recovery [4], reverse slowed action potential conduction, shorten action potential duration, and improve myocardial contractility in the face of increased plasma concentrations of bupivacaine [5].

Accidental intravascular injection of bupivacaine, over-absorption from peripheral tissues, and high plasma concentrations contribute to life-threatening cardiac rhythm disturbances. Previous studies suggested that the mechanism of bupivacaine-induced cardiac toxicity was secondary to its depression of the cardiac conduction system [6] through inhibition of the sodium channels [7,8] potassium channels [9,10] and calcium channels [11,12].

We hypothesized that epinephrine can reverse bupivacaine-induced cardiac inhibition by modulating I_Na,_ I_Ca-L_, and I_to_ membrane channels of ventricular myocytes. We used the whole-cell patch clamp technique to observe the effect of epinephrine on these membrane channels in rat ventricular myocytes that were subjected to high-concentration bupivacaine-induced cardiac toxicity.

## Methods

### Isolated heart and preparation

Ths study was approved by the Animal Care and Use Committee of Wenzhou Medical College. Adult Male Sprague-Dawley rats, weighing 250 to 300 g, were anesthetized using 5% chloral hydrate (7 ml/kg intraperitioneal injection). The carotid artery is cut to allow rapid and thorough exsanguination. The hearts were rapidly removed intact with a short aortic remnant. While in this Tyrode solution the aorta is cannulated, one should make sure that there are no air bubbles trapped in the cannula. The cannulated heart is then mounted on a Langendorff perfusion apparatus with constant flow. After rapid excision, the heart was mounted in a modified Langendorff system and then perfused with the nominally Ca2 + -free Tytode solution (NaCl 137 mM、KCl 5.4 mM、MgCl_2_ 1.0 mM、NaH_2_PO_4_ 0.33 mM、HEPES 10 mM、Glucose 10 mM, and PH7.35 with NaOH) for 5 min at room temperature (20-25)°C. The perfusate reservoirs and column are glass jacketed, allowing the temperature to be maintained at 37°C by means of a recirculating water bath. All of the perfusion solutions are equilibrated with 100% oxygen. The heart is then retrogradely perfused with the nominally Ca2 + -free Tyrode solution which causes cessation of the heartbeat until the blood is washed out. This was followed by perfusion with enzyme solution, containing 1 mg/ml collagenase (Sigma, typeI) in nominally Ca2 + -free Tytode solution, followed for 10 ~ 15 min. The softened heart was removed from the column, and the left ventricle was dissected in modified KB (KCl 40 mM, KH_2_PO_4_ 20 mM, MgSO_4_ 3.0 mM, KOH 80 mM, Glutamate 50 mM, Taurine 20 mM, HEPES 10 mM, glucose 10 mM, EGTA 0.5 mM, and pH 7.35 with KOH). The cells were maintained in modified KB solution and stabilized at room temperature for 1 hour.

### Equipments and methods

An EPC-10 patch clamp amplifier (HEKA, Germany) was used for application of the whole-cell patch clamp in the ventricular myocytes. Pulse stimulation and data acquisition were recorded by Pulse 8.0 software (HEKA, Germany). Patch pipettes were pulled from glass tubing with a 1.5 mm outer diameter (SUTTER, USA) by the use of micro-electrodes (P-97, SUTTER, USA), and the tip was heated to give a resistance of 1.5-2.5 MΩ when filled with the specific, appropriate solution (see below). Using an inverted microscope, microelectrodes were directed to the ventricular myocytes by a three-dimensional micromanipulator (MP-285, SUTTER, USA). A Giga-seal was formed after vacuum suction. The patch membrane was broken after fast capacitance compensation by the provision of additional suction with subsequent construction of a whole-cell recording. To reduce the instantaneous current charging/discharging, and to minimize clamping errors, the slow capacitance compensation and series resistance compensation were settled at 70% - 80%. Leakage currents were subtracted by the P/4 method.

### Electrophysiology

1. For recording INa, the external solution was composed of the following, in mM: Choline-Cl 120, NaCl 20, MgCl2 1.0, HEPES 5, Glucose 10, CsCl 4.6, pH 7.35 with CsOH. the internal solution was composed of the following, in mM: CsCl 120、NaCl 10、MgCl2 1.0、Na2ATP 5.0、EGTA 10、HEPES 10, pH 7.3 with CsOH.

The potential was held at -90 mV, I_Na_ was evoked by 25 ms, and accompanied by -30 mV square-wave depolarizing pluses. The stimulation program of current density-voltage curve was as follows: under a holding potential (Vh) of -90 mV, the step clamp voltage (Vs) was stimulated from -90 mV to +50 mV by a step of 10 mV with a 50 ms duration, and a 0.2 Hz stimulation frequency.

2. For recording I_Ca-L_, the external solution was composed of the following, in mM: Choline-Cl 140, MgCl2 1.0, CaCl2 2.0, HEPES 5, Glucose 10, CsCl 4.6, TEA-Cl 10, pH 7.35 with CsOH. the internal solution was composed of the following, in mM: CsCl 120、MgCl_2_ 1.0、MgATP 5.0、EGTA 10、HEPES 10 TEA-Cl 10, pH 7.3 with CsOH.

The potential was held at -40 mV, and I_Ca-L_ was evoked by 150 ms, accompanied by 0 mV square-wave depolarizing pluses. The stimulation program of current density-voltage curve was as follows: under a Vh of -40 mV, the Vs was stimulated from -40 mV to +50 mV by a step of 10 mV with a 250 ms duration, and a 0.2 Hz stimulation frequency.

3. For recording I_to_, the external solution was composed of the following, in mM: NaCl 137, KCl 5.4, CaCl2 1.8, MgCl2 1.0, NaH2PO4 0.33, HEPES 10, Glucose 10, CdCl2 0.3, pH t7.35 with NaOH.the internal solution was composed of the following, in mM: KCl 140、MgCl_2_ 1.0、K_2_ATP 5.0、EGTA 10、HEPES 5,pH 7.3 with KOH.

The potential was held at -90 mV, I_to_ was evoked by 20 ms, 50 mV square-wave depolarizing pluses. The stimulation program of current density-voltage curve was: under a Vh of -90 mV, and I_Na_ was eliminated by 20 ms, with -40 mV depolarizing pluses; the Vs was stimulated from -40 mV to +50 mV by a step of 10 mV with a 400 ms duration and a 0.2 Hz stimulation frequency.

A control curve of I-V was collected before drug perfusion (T0). Then 100 umol/L bupivacaine and the mixture of 100 umol/L bupivacaine and 0.15 μg/ml epinephrine were added into the reservoirs respectively from superfusion systerm (DADVC-8PP,ALA SCIENTIFIC, USA). The DAD-VC systems go out with a Micromanifold consisting of 8 tubes of polyamide coated quartz glass of 100 um ID. The Micromanifold enables up to 8 solutions from the reservoirs to flow into a small common space of less than 1ul. The Micromanifold with a micromanipulator can easily be moved around the cell preparation and pointed at the target cell. The user must be careful to aim the output so that it completely bathes the cell under study. So during the study there is no motion of the output tip to be dealt with and there is no need to have all the solutions flowing out and contaminating the preparation solution during an experiment. After the cell surface perfusion with 100 μmol/L bupivacaine for 10 seconds. The peak current and I-V curve were recorded (T1). The microperfusion tube was then swithed by another reservoir prefilled with 100 μmol/L bupivacaine and 0.15 ug/ml epinephrine after the model was successfully made by 100 μmol/L bupivacaine. The peak current and I-V curve were then recorded at the time (T2) when the cells’ surface were perfusion with 100 μmol/L bupivacaine and 0.15 ug/ml epinephrine for 10 seconds.

To eliminate the error among cells, the size of the ion currents was represented by the current density, which was the ratio of current intensity and cell membrane capacitance (pA/pF). Data were stored in the hard disk for the measurement and analysis. Raw current data were analyzed and measured by pulse 8.0.

### Statistical analysis

All data were analyzed using SPSS 13.0 and presented as mean ± SD. The results were analyzed with a paired *t* test, the P value were using the Bonferroni correction,. A *P* value < 0.05 was considered statistical significance. The graphs were performed by Origin 8.0.

## Results

The various ion currents could be obtained from the fresh isolated ventricular myocytes, which indicated that the cells had good electrophysiological properties.

### Effects of epinephrine on bupivacaine-induced inhibition of sodium currents in ventricular myocytes of rats

**Figure 1:**
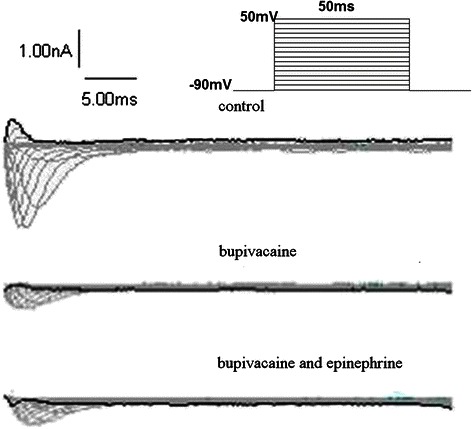
Representative I_Na_ traces obtained from ventricular myocytes groups: control, bupivacaine 100 μmol/L alone, and bupivacaine with epinephrine 0.15 μg/ml. Family of current traces obtained by applying a series of 50 ms pulses ranging from -90 mV to 50 mV with a holding potential of -90 mV.

Sodium currents: The I_Na_ at T_0_, T_1_, and T_2_ was (-8.3 ± 0.9) (pA/pF), (-2.2 ± 0. 6) (pA/pF), and (-2.3 ± 0.7) (pA/pF), respectively (n = 5, T_1_*vs* T_0_, *P* < 0.001; T_2_*vs* T_0_, *P* < 0.001; T_2_*vs* T_1_, *P* =0.322) (Table 1). Figure 1 depicts the effects of bupivacaine alone and in combination with epinephrine on the I_Na_ trace.

**Table 1:** **Bupivacaine alone and combination with epinephrine affect the channels current-density**

Channel current	Cell number	TO (pA/pF)	T1 (pA/pF)	T2 (pA/pF)
I_Na_	5	−8.3 ± 0.9	−2.2 ± 0. 6	−2.3 ± 0.7
I_Ca-L_	6	−7. 8 ± 0.7	−2.0 ± 0.6	−4.9 ± 0.9
I_to_	6	23 ± 5	15 ± 3	26 ± 8

Data were shown as mean ± SD. T0 = before the bupivacaine perfusion; T1 = after the perfusion of the 100 μmol/L bupivacaine; T2 = after the perfusion of the 100 μmol/L bupivacaine with 0.15 μg/ml epinephrine.

I_Na_: T1 vs T0, *P* < 0.001; T2 vs T1, *P* >0.05.

I_Ca-L_: T1 vs T0, *P* < 0.001; T2 vs T1, *P* < 0.001.

I_to_: T_1_*vs* T_0_, *P* < 0.05; T_2_*vs* T_1_, *P* < 0.05.

**Figure 2:**
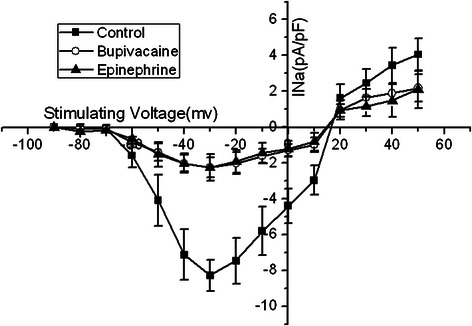
The I_Na_ current-voltage relationship in the absence of bupivacaine, with bupivacaine 100 μmol/L alone, and bupivacaine 100 μmol/L in combination with epinephrine 0.15 μg/ml. Bupivacaine 100 μmol/L present in the perfusate contributed to an upper shift of the current density-voltage curve. Administration of 100 μmol/L bupivacaine and epinephrine 0.15 μg/ml did not effect any change in the curve.

Sodium channel current density-voltage curve: The activation potential of I_Na_ was -60 mV, with a peak potential of -30 mV and a reverse potential of +20 mV. Bupivacaine 100 μmol/L present in the perfusate lead to the I_Na_ decrease, and an upper shift of the current density-voltage curve, without changing the peak potential, activation potential, or the reversal potential. Administration of bupivacaine 100 μmol/L and epinephrine 0.15 μg/ml did not effect any change in the I_Na_ current density-voltage curve (Figure 2).

### Effects of epinephrine on bupivacaine-induced inhibition of L-type calcium currents in ventricular myocytes of rats

**Figure 3:**
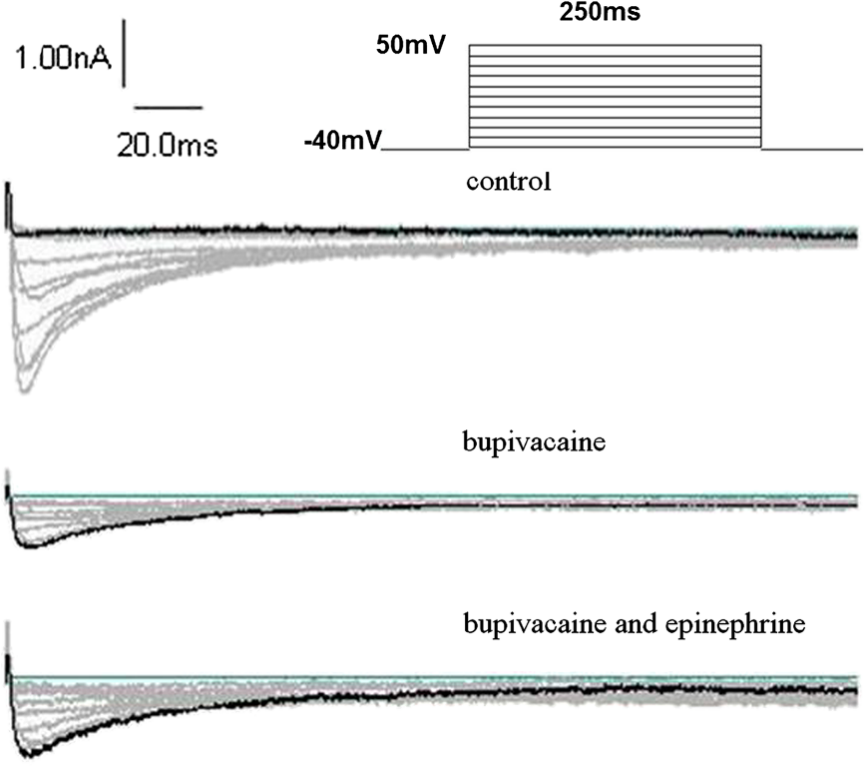
Representative I_Ca_ traces obtained from ventricular myocytes groups: control, bupivacaine 100 μmol/L alone, and bupivacaine 100 μmol/L in combination with epinephrine 0.15 μg/ml. Family of current traces obtained by applying a series of 250 ms pulses ranging from -40 mV to 50 mV with a holding potential of -40 mV.

L-type calcium currents: The I_Ca-L_ at T_0_, T_1_, and T_2_ was (-7.8 ± 0.7) (pA/pF), (-2.0 ± 0. 6) (pA/pF), and (-4.9 ± 0.9) (pA/pF), respectively (n = 6, T_1_*vs* T_0_, *P* < 0.001; T_2_*vs* T_0_, *P* < 0.001; T_2_*vs* T_1_, *P* < 0.001). Figure 3 depicts the effects of bupivacaine 100 μmol/L alone and in combination with epinephrine 0.15 μg/ml on the I_Ca-L_ trace.

**Figure 4:**
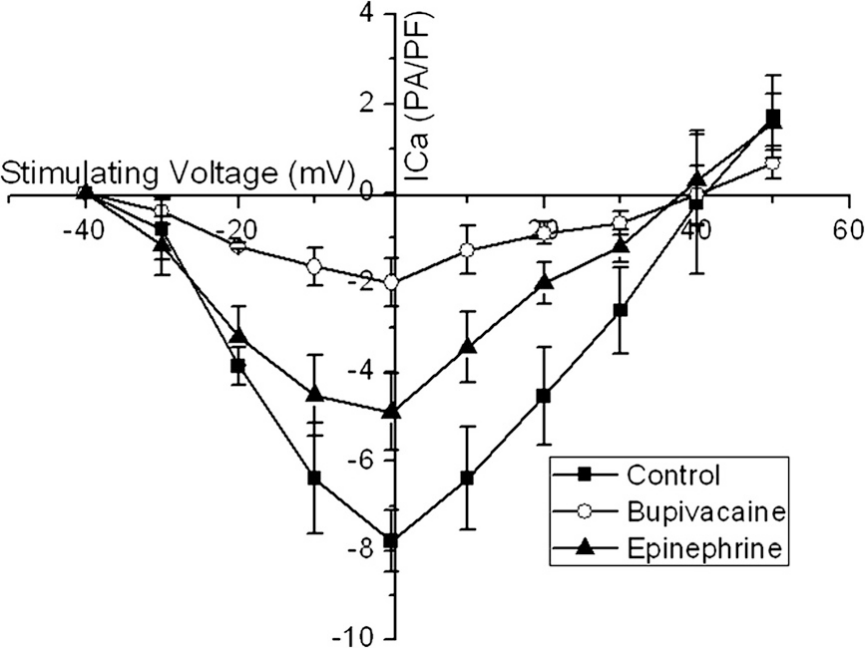
The I_Ca-L_ current-voltage relationship in the absence of bupivacaine, with bupivacaine 100 μmol/L alone, and bupivacaine 100 μmol/L in combination with epinephrine 0.15 μg/ml. Bupivacaine 100 μmol/L present in the perfusate contributed to an upper shift of the current density - voltage curve without changing the activation potential and the curve shape. However, bupivacaine 100 μmol/L in combination with epinephrine 0.15 μg/ml increased the I_Ca-L_ current and moved the curve of I_Ca-L_ downward.

I_Ca-L_ was -30 mV, with a peak potential of 0 mV and a reverse potential of +40 mV. Bupivacaine 100 μmol/L present in the perfusate contributed to the upper shift of the current density - voltage curve without changing the activation potential and the curve shape. However, bupivacaine 100 μmol/L in combination with epinephrine 0.15 μg/ml increased the I_Ca-L_ current, and moved the current density-voltage curve of I_Ca-L_ downward (Figure 4).

### Effects of epinephrine on bupivacaine-induced inhibition of transient outward potassium currents in ventricular myocytes of rats

**Figure 5:**
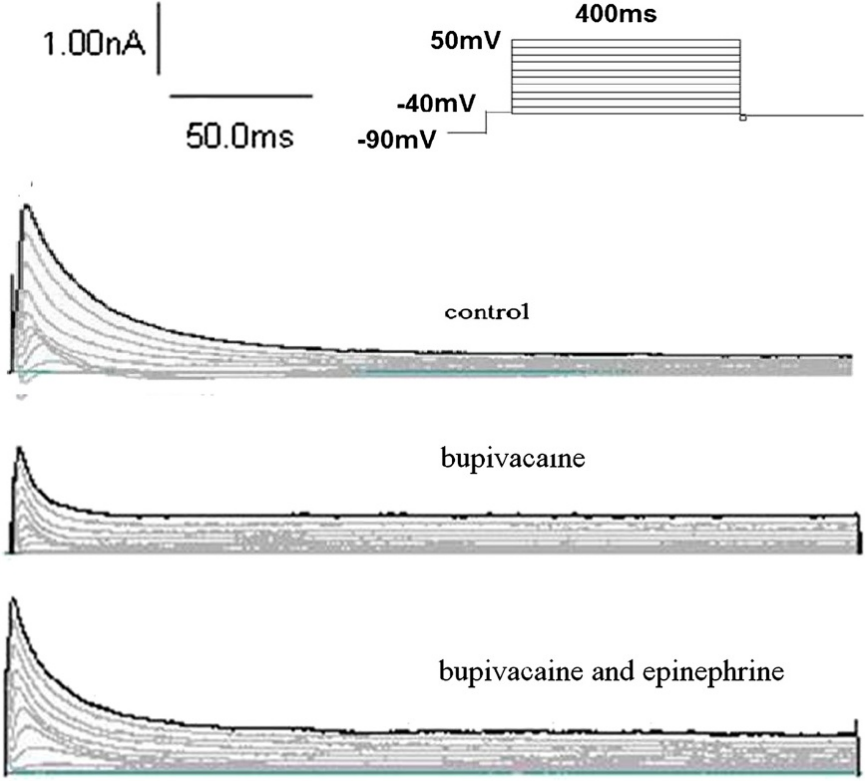
Representative I_to_ traces obtained from ventricular myocyte groups: control, bupivacaine 100 μmol/L alone, and bupivacaine 100 μmol/L in combination with epinephrine 0.15 μg/ml. Family of current traces obtained by applying a series of 20ms test pulses of -40 mV to eliminate current through Na channels, which was then evoked by a series of 400 ms pulses ranging from -40 mV to 50 mV with a holding potential of -90 mV.

Transient outward potassium currents: The current density of I_to_ at T_0_, T_1_, and T_2_ was (23 ± 5) (pA/pF), (15 ± 3) (pA/pF), and (26 ± 8) (pA/pF), respectively (n = 6, T_1_*vs* T_0_, *P* =0.013; T_2_*vs* T_0_, *P* = 0.161; T_2_*vs* T_1_, *P* =0.003). Figure 5 depicts the effects of bupivacaine 100 μmol/L alone, and in combination with epinephrine 0.15 μg/ml on the I_to_ trace.

**Figure 6:**
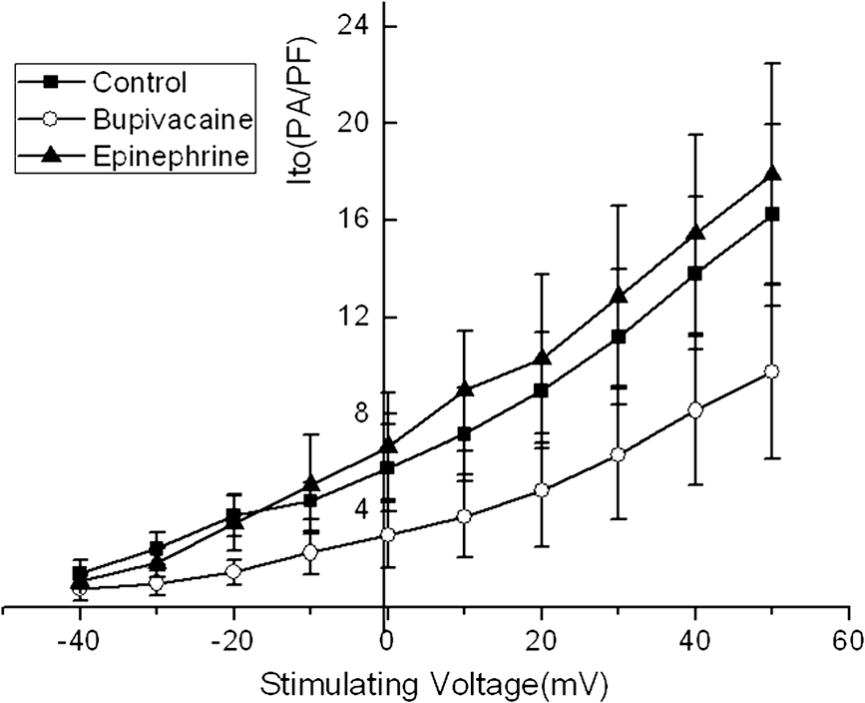
The I_to_ current-voltage relationship in the absence of bupivacaine, with 100 μmol/L bupivacaine alone, and 100 μmol/L bupivacaine in combination with epinephrine 0.15 μg/ml. Bupivacaine 100 μmol/L without epinephrine decreased I_to_. On the other hand, I_to_ increased in response to the bupivacaine 100 μmol/L in combination with epinephrine 0.15 μg/ml.

Transient outward potassium channel currents density-voltage curves: The activation potential of I_to_ was at -40 mV with the higher voltage the current was increased, and the outward rectification characteristics were evident. Bupivacaine 100 μmol/L without epinephrine decreased I_to_. On the other hand, I_to_ increased in response to the bupivacaine 100 μmol/L in combination with epinephrine 0.15 μg/ml. However, both their rectifier characteristics were not changed (Figure 6).

## Discussion

This study demonstrates that epinephrine can reverse bupivacaine-induced inhibition of calcium channels and the transient outward current of the potassium channels in the ventricular myocytes of rats, but with no significant effect on improving ionic flow in the sodium channels, i.e., reversing its inhibition.

Epinephrine is a potent heart stimulant. Currently, the American Heart Association recommends use of epinephrine during CPR [13], as a first-line drug for treating cardiac arrest, however, the use of epinephrine for bupivacaine-induced cardiac toxicity remains controversial [1-4].

The essence of bupivacaine-induced cardiac toxicity involves its ability to block myocardial cell ion channels. Rapid intravenous injection of a clinical dose of bupivacaine may lead to a cardiac arrest secondary to high plasma concentrations of bupivacaine that reach ≥ 100 μmol/L in a short period of time [14]. Thereby it reducing the amplitude of the myocardial action potential, shortening the action potential duration [12], and significantly inhibiting the myocardial sodium [6], potassium [8] and calcium [11] channel function. Moreover, while our results confirm these findings, we also have electrophysiological confirmation that epinephrine alone may be of little use in regard to sodium channel “resuscitation” as opposed to its ability to aid the calcium or potassium channels. Discussion of our study’s impact on the sodium, calcium, and potassium channels is warranted at this juncture.

I_Na_ facilitates the depolarization of the action potential (phrase 0), which affects excitability, refractoriness and conductivity of myocardial cells. Depolarizing of this action potential is impeded by bupivacaine, and its cardiac toxicity is directly related to the impaired flow Na^+^ inflow, which results in conduction disturbances and a series of cardiac arrhythmias [6] that may lead to cardiac arrest. A plasma bupivacaine concentration of 100 μmol/L blocks 73% of the sodium current. Catecholamine impact on the Na^+^ currents remains controversial. Wang [15] and Gintant [16] indicate that β-receptor agonists increase the sodium current, while others report that high concentrations of isoproterenol (a ß agonist) can produce inhibition of Na^+^ currents [17]. Our results indicate that epinephrine does not effectively support the recovery of ionic flow in the bupivacaine-inhibited sodium channels. This suggests that the use of epinephrine in reversing bupivacaine-induced cardiac toxicity may have limitations.

I_Ca-L_ plays an important role in the formation of the action potential plateau by increasing intracellular calcium and myocardial contraction. There is a concentration-dependent relationship between bupivacaine and its inhibition of L-type calcium channels in rat ventricular myocytes. A bupivacaine concentration of 100 μmol/L is roughly equivalent to the IC_50_ inhibition of I_Ca-L_ [11]. The inhibitory effect of I_Ca-L_ in our study was 50% higher than that reported in the literature and this may be related to our method of drug perfusion. We ensured that the bupivacaine concentration on the surface of the cell membrane reached 100 μmol/L instantly by using a direct microperfusion system. The cells in this case lacked a process of gradual adaptation, which may have enhanced the inhibitory effect on the channels. This situation may be analogous with the clinical picture of acute bupivacaine toxicity. Although β-adrenergic agonists can augment the frequency of ionic channel opening and current passage through the L-type calcium channel [18,19], there are no published reports as to whether epinephrine can reverse bupivacaine-induced inhibition of calcium channels. In our study bupivacaine suppressed the calcium current, but this current was partially restored by epinephrine. Thereby contributing to improved myocardial contractility and physiologic recovery.

With the rapid activation and inactivation in the early repolarization period of the action potential, I_to_ exhibited a concentration-dependent inhibition by bupivacaine on ventricular myocytes [8]. In humans, the inhibition of I_to_ prolongs the repolarization of the myocardial action potential and reduces the excitation-contraction coupling efficiency of the myocardial cells. Thus weakening the cardiac contractility by impairing regulation of the sarcoplasmic reticulum’s calcium load, releasing and influencing the function of the L-type calcium channel, and impairing the sodium calcium exchange mechanism. Additionally, the I_to_ of rat ventricular myocytess can be partially inhibited by α_1_-adrenergic receptor agonists [20]. However, our study demonstrates that epinephrine can reverse the inhibition of I_to_ induced by bupivacaine. Through the enhancement of the calcium current and an increased intracellular Ca^2+^ concentration secondary to activated β adrenergic receptors, there is a resultant increase in cardiac contractility through the indirect recovery of calcium-dependent I_to_ [21].

In this study we used the dose of epinephrine 0.15 μg/ml, which was based on our previous work demonstrating that isolated, arrested rats hearts recovered with this particular dose [22]. Additionally, we added bupivacaine 100 μmol/L to avoid bupivacaine elution in the process of extracellular fluid perfusion on the cell’s surface in order to simulate a setting where there is a critical plasma concentration of bupivacaine that induces cardiac toxicity.

The results/conclusions of our study are limited by the fact that we did not observe any reversal effect of epinephrine on the inhibition of sodium channel induced by different concentrations of bupivacaine, and we did not observe any reversal of the inhibitory effect of differing concentrations of epinephrine on the ion channels.

## Conclusions

Epinephrine can reverse high-concentration bupivacaine-induced inhibition of calcium channels and the transient outward potassium current channels in the ventricular myocytes of rats, whereas it has no significant effect on improving sodium channel ionic flows. Thereby providing no augmentation of its action potential depolarization. These findings support the position that the use of epinephrine alone in the resuscitation of bupivacaine-induced cardiac toxicity/arrest may be of limited value in that it may produce a less than optimal result. It may be that adjunctive therapies/medications are needed to augment the use of epinephrine in the setting of bupivacaine-induced cardiac toxicity/arrest.

## Abbreviations

KB: Kraft-Bruhe
4-AP: 4-Aminopyridine
TEA-Cl: Tetraethylammonium chloride
BSA: Bovine Serum Albumin
HEPES: N-2-hydroxyethylpiperazine-N-ethane-sulphonicacid
I_Ca-L_: L-type calcium current
I_Ca-T_: T-type calcium current
I_Na_: Sodium current
I_K1_: Inward rectifier potassium current
I_to_: Transient outward potassium current
V_h_: Membrane holding potential
V_rev_: Reverse potential
Vs: Step holding potential
IC50: Half-maximal inhibitory concentration
